# An ALMS1 variant disrupts proximal centriole organization and promotes a myofibroblast-like phenotype that is regulated by THY1

**DOI:** 10.21203/rs.3.rs-10872429/v1

**Published:** 2026-09-15

**Authors:** Angela Zeigler, Sarah Colijn, Ankur Gholkar, Song Yang, Xuedong Kang, Yan Zhao, Charlotte Wolf, Song Li, Jorge Torres, Stan Nelson, Amber Stratman, Marlin Touma

**Affiliations:** UCLA David Geffen School of Medicine; Washington University School of Medicine; University of California, Los Angeles, CA.; University of California, Los Angeles, CA.; University of California, Los Angeles; University of California, Los Angeles; University of California, Los Angeles; University of California, Los Angeles; University of California Los Angeles; David Geffen School of Medicine, UCLA; Washington University School of Medicine; David Geffen School of Medicine, University of California, Los Angeles, CA.

**Keywords:** ALMS1, THY1, endocardial fibroelastosis, cilia, STED imaging, Rootletin, fibroblast

## Abstract

Primary endocardial fibroelastosis (pEFE) is characterized by accumulation of elastin-rich extracellular matrix (ECM) in the left ventricular endocardium in the absence of structural defects, but its pathophysiology remains unclear. We investigated dermal fibroblasts from a pEFE patient (proband) harboring a loss-of-function ALMS1 variant. Compared with control fibroblasts, proband cells displayed a myofibroblast-like phenotype, with increased ECM proteins and markers of fibroblast activation and endothelial-to-mesenchymal transition. Conversely, the thymus cell surface antigen (THY1) was downregulated in the proband fibroblasts, which also exhibited reduced ciliation. STED microscopy revealed ALMS1 as a concave, cap-like structure extending into the proximal end of the centriole lumen in control fibroblasts, while it was fragmented in proband fibroblasts and associated with reduced and disorganized Rootletin and C-NAP1 proteins and abnormal centriole separation. Importantly, soluble THY1 reduced production of matrix proteins and markers of fibroblast activation without effects on centriole organization or ciliation. These findings indicate that ALMS1 dysfunction contributes to pEFE pathology through both abnormal proximal centriole organization and THY1-negative fibroblast activation, implicating THY1 as a potential target for pEFE therapy.

## 1. INTRODUCTION

Endocardial fibroelastosis (EFE) is a severe prenatal or perinatal cardiac pathology ^1–4^ characterized by significant endocardial thickening that results in cardiac dysfunction and early death. Generally, it is classified into primary EFE (pEFE) and EFE associated with structural heart disease (secondary EFE), although it is not entirely clear in some cases whether the structural lesion is a cause or effect of the fibrosis ^1–3^.

The involvement of the endocardium in pEFE pathology is a unique phenomenon. Specifically, a thick elastin-rich fibrotic layer accumulates in the sub-endocardium of the left ventricle (LV), restricting the myocardium initially and ultimately causing LV dilation and end-stage heart failure, leading to death or heart transplantation ^1–3^. Unlike secondary EFE, which is commonly seen in left-sided obstructive defects such as hypoplastic left heart syndrome (HLHS) where abnormal flow patterns play key roles ^3^, no structural defect is identified in pEFE cases ^2^. Fibroelastosis is a specialized form of fibrosis that is thought to be driven by TGFβ-mediated induction of endothelial-to-mesenchymal transition (endoMT) ^4,5^. This leads to an increase in activated fibroblasts (myofibroblasts), which are characterized by excessive production of elastin, fibrillin, and collagen, thereby driving significant extracellular matrix (ECM) remodeling. However, how these cells arise, where they originate, and the exact pathophysiology driving the development of pEFE remain under active investigation ^4–6^.

We recently carried out genomic studies on pEFE-affected families and identified a previously unreported association between pEFE and loss-of-function variants in *ALMS1*, which encodes the centrosome and basal body-associated protein 1 ^7^. ALMS1 is a structural protein localized to the proximal end of the centriole and has been implicated in ciliogenesis, centrosome organization, and cell division^7–11^. Primary cilia and the centrosome play essential roles in cardiac development, acting as signaling hubs that influence cardiomyocyte and fibroblast differentiation ^12,13^. Importantly, ciliary defects are known to be associated with cardiac fibrosis and congenital heart disease ^14–16^. Previously reported *ALMS1* variants have been shown to cause Alström syndrome (AS) [OMIM: 203800], a rare recessive ciliopathy, which typically manifests with severe multi-organ dysfunction during early childhood^7–11,17–19^.

Our previous work established the first link between pEFE and cilia/centrosome dysfunction^7^, yet how ALMS1 loss drives fibroelastosis remains unclear, meriting further investigation. Importantly, ALMS1 function in ciliogenesis remains incompletely understood, as studies have reported different consequences linked to loss-of-function variants versus genetic knockout models. RNAi-mediated depletion of *ALMS1* produces profound ciliogenesis defects ^8,9,11^, whereas patient-derived cells with pathogenic *ALMS1* variants often retain the ability to assemble primary cilia ^10,18,19^. This suggests that the consequences of ALMS1 loss are context-dependent and highlights patient-derived cells as a physiologically relevant model for investigating disease mechanisms linked to individual human variants.

Here, we employ fibroblasts derived from our previously reported pEFE patient with a homozygous loss-of-function *ALMS1* variant ^7^. The proband initially presented with isolated end-stage heart failure requiring heart transplantation before subsequently developing progressive cone-rod dystrophy leading to blindness, neuronal hearing loss, early-onset obesity, type 2 diabetes, and short stature, all of which are known clinical features of AS ^7–11,17–19^. Using our proband-derived cells ^7^, we show that ALMS1 loss decreases fibroblast ciliation and induces a myofibroblast-like phenotype, consistent with the hallmarks of pEFE fibrosis. Mechanistically, we show using stimulated emission depletion (STED) super-resolution microscopy that ALMS1 loss disturbs centriole cohesion by disrupting Rootletin fibers, thereby increasing centriole separation. Finally, transcriptomic analyses revealed downregulation of thymus cell antigen (*THY1*) in proband fibroblasts and we observed that adding soluble THY1 (sTHY1) was able to reduce Elastin and markers of fibroblast activation and rescue the myofibroblast-like phenotypes, indicating a potential role for THY1 in regulating and mitigating the pathophysiology of pEFE.

## 2. METHODS

### 2.1 Cell culture and treatment

The *ALMS1*-variant neonatal dermal fibroblasts derived from the pEFE proband and control human neonatal dermal fibroblast line (hNDFs; ATCC) were maintained on 1 mg/mL gelatin-coated dishes in DMEM (#11995–065; Gibco) supplemented with 10% FBS (Gibco) and 1× antibiotic-antimycotic (#15240–062; Gibco). Cells were used up to passage 8. DMEM plus 1x Anti-Anti without FBS was used for serum starvation. Serum activation was achieved by serum-starving cells for 48 hours, then adding growth media for 24 hours. For soluble THY1 (sTHY1) experiments, cells were plated in either growth serum or serum-starved media, then treated with sTHY1 recombinant protein (1 μg/mL; #10805-TH; R&D Systems) or vehicle control (PBS) for 48 hours.

Human umbilical vein endothelial cells (HUVECs; ATCC) were grown as previously described ^20^. Plated and methanol-fixed retinal pigment epithelial cells (RPE1) were provided by Jennifer Wang (Washington University in St. Louis) ^21^.

### 2.2 Immunocytochemistry

For imaging, control and proband cells were plated on #1.5 coverslips (#64–0713; Warner Instruments) and treated as described above. Cells were then fixed with 4% PFA or ice-cold methanol for 15 minutes, then rinsed 3x with PBS. PFA-fixed cells were then permeabilized with 0.5% TritonX100 for 20 minutes, then rinsed 3x with PBS. Cells were then blocked [3% BSA, 22.52 mg/ml glycine in PBS with 0.1% Tween 20 (PBST)] for 30 minutes and incubated with primary antibody diluted in 1% BSA in PBST for 1 hour. After washing 3x with PBS, slides were incubated in secondary antibody in 1% BSA in PBS for 1 hour. After final wash, coverslips were mounted on glass slides and imaged. For fluorescence images, ProLong Gold Antifade mounting solution (Thermo Fisher Scientific) was used.

Primary antibodies and dyes used for immunocytochemistry are listed in Table 1. Secondary antibodies from Invitrogen and Abberior Instruments, shown in Table 1, were used for immunofluorescence microscopy, confocal imaging, and STED imaging, respectively. Hoechst 34580 (Sigma-Aldrich) was used as a nuclear counterstain. Mounting media used were: Fluoromount Aqueous Mounting Medium (#F4680, Sigma-Aldrich) for confocal and Abberior Mount, Liquid Antifade (#MM-2009, Abberior Instruments) for STED.

### 2.3 Image Acquisition and Processing

Immunofluorescence Microscopy images were captured with a Leica DMI6000 microscope (Leica Microsystems, 63x/1.40 NA oil objective, Leica Application Suite AF6000 software) and exported as TIFF files. Confocal images were obtained using a 20x, 40x water-immersion, or 60x oil-immersion objective with a W1 Spinning Disk confocal microscope, a Fusion camera, and the Nikon Eclipse Ti2-E base. Images were then deconvolved using Nikon’s NIS-Elements software. High-resolution images were obtained using a 100x high NA oil-immersion objective with a Zeiss LSM 880 Confocal with Airyscan. Images were then deconvolved using ZEISS ZEN software. Super-resolution images were obtained using a 100x/1.4 NA oil-immersion objective with a Leica DMi6000 inverted microscope equipped with an Abberior STEDYCON 2D system. Depletion was achieved using a 775 nm pulsed STED laser. Image acquisition was controlled via the Abberior browser-based interface. Images were then deconvolved using Huygens Essential software (Scientific Volume Imaging, Hilversum, Netherlands). Fiji image processing software was used for image analysis.

### 2.4 Immunofluorescence Imaging Quantification and Analyses for Spindle Length.

**Figure 1:**
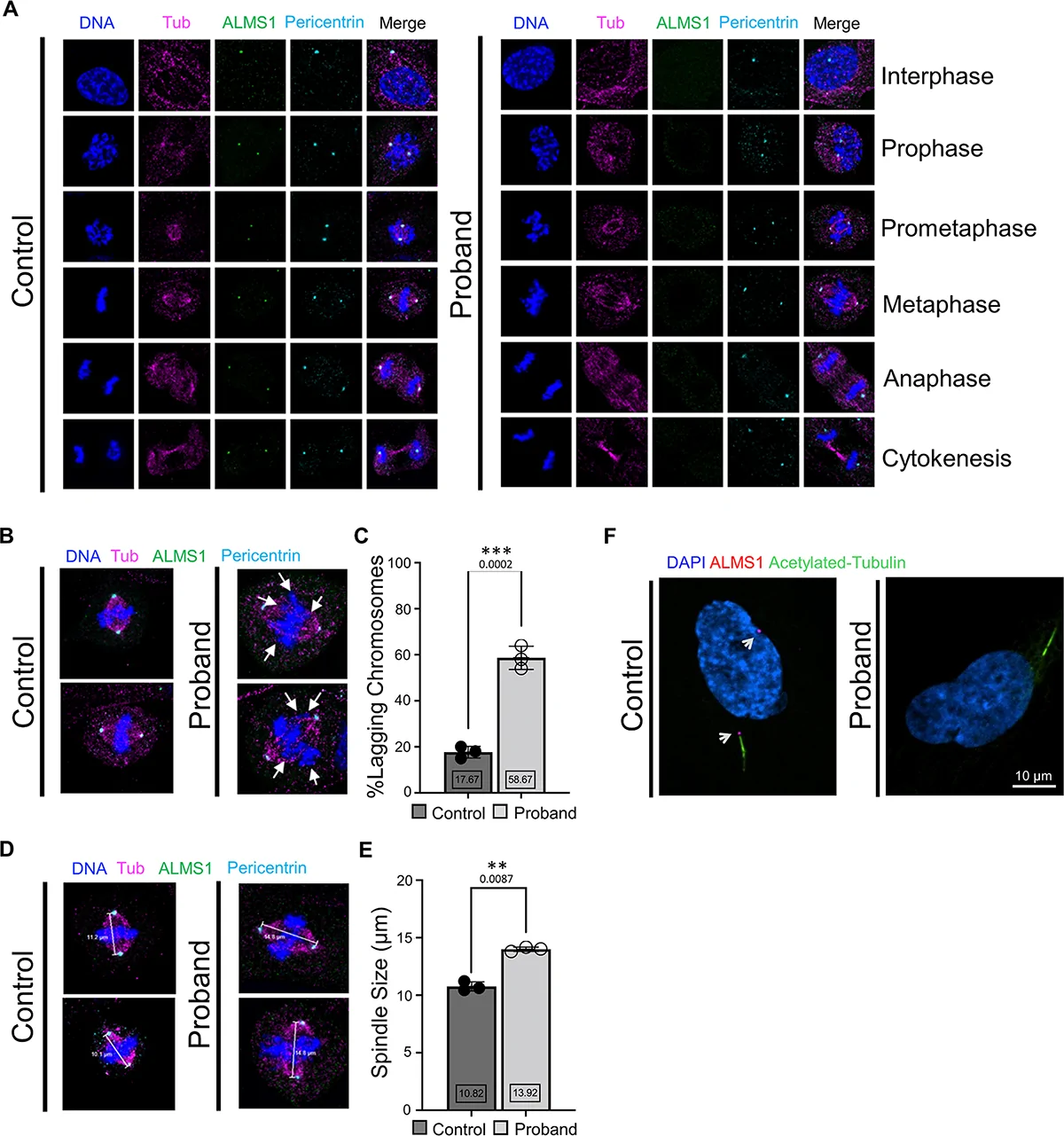
ALMS1 is depleted in proband fibroblasts. **(A)** Immunostaining of ALMS1 in control human neonatal dermal fibroblasts (hNDFs) and *ALMS1*-variant proband fibroblasts during mitosis. Control hNDFs display ALMS1 localization at the centrioles throughout cell cycle phases. In contrast, ALMS1 is not detectable at the centrioles in proband cells. (**B-E)** Immunostaining of mitotic spindles during metaphase in control hNDFs and proband fibroblasts. Proband fibroblasts exhibit chromosomal lagging at the central plate (**B**) and spindle elongation (**D**) during metaphase, as quantified in (**C**) and (**E**), respectively. N=3 independent experiments. **(F)** Immunostaining of ALMS1 in control hNDFs and proband fibroblasts after 48 hours of serum starvation. Control hNDFs display ALMS1 localization at the basal body of cilia. In contrast, ALMS1 is not detectable at the basal body of cilia in proband cells. Scale bars: 20 μm (A), 10 μm (B,D,F). Statistics for panels C and E were calculated using Student’s t-tests. Data are presented as the mean ± S.D

For each condition, 15 spindles were measured pole-to-pole with pericentrin as a reference, per experiment i.e. 45 spindle measurements in total within 3 independent experiments using Leica AF6000/LAS-X software. For percent of cells with lagging chromosomes, for each condition, a total of 100 cells were observed, per experiment i.e. 300 cells in total across 3 independent experiments using Leica AF6000/LAS-X software. Data was judged statistically significant when *P* < 0.05. Asterisks indicate statistical significance; * *P* < 0.05, ** *P* < 0.01, *** *P* < 0.001. Data graphs in Fig. 1 were generated with GraphPad Prism 5 and are presented as the mean ± SD.

### 2.5 qPCR Analysis

Total RNA was isolated from the cultured cells using the PureLink^™^ RNA Mini Kit (Thermo Fisher Scientific, 12183025). The reverse transcription reaction was performed with 1 μg of total RNA and the High-Capacity cDNA Reverse Transcription Kit (Thermo Fisher Scientific, 4368814), and the real-time PCRs were performed using iTaq Universal SYBR Green Supermix (BioRad, 1725121). Primers were prepared by Integrated DNA Technologies. The relative expression value was calculated using the comparative threshold cycle (ΔΔCT) method.

### 2.6 ELISA Assays

Protein concentrations were quantified using a sandwich enzyme-linked immunosorbent assay (ELISA) according to the manufacturer’s instructions. Briefly, samples and standards were added to antibody-coated 96-well plates and incubated with a biotinylated detection antibody followed by horseradish peroxidase (HRP)-conjugated streptavidin. Color development was achieved using 3,3′,5,5′-tetramethylbenzidine (TMB) substrate and stopped with sulfuric acid. Absorbance was measured at 450 nm using a microplate reader, and protein concentrations were calculated from a standard curve.

ELISA experiments were conducted using the TGF-β1 ELISA kit, Invitrogen (Cat# BMS248–4) 96 tests, the Abcam Human Elastin ELISA kit (Cat# ab239433) 96 tests, the COL25A1 ELISA kit, Mybiosource (Cat#MBS9316392) 48 tests, and the COL5A3 ELISA Kit Mybiosource (Cat# MBS7242752) 48 tests.

### 2.7 Bulk RNA sequencing

Total RNA isolated from *ALMS1*-variant dermal fibroblasts derived from the pEFE proband and control human neonatal dermal fibroblasts (hNDFs) obtained from ATCC were processed for bulk RNA sequencing with three technical replicates from each, as was previously described ^7^. Briefly, samples were processed with the Illumina Paired-End Sample Prep Kit, and quantification was performed on the Agilent 2100 Bioanalyzer and ABI StepOnePlus Real-Time PCR system. Libraries were sequenced using the Illumina HiSeqTM 400 sequencer protocol and aligned to a reference human genome using TopHat.

### 2.8 Bulk RNA sequencing analysis

The raw unnormalized bulk RNA-seq datasets were re-analyzed using DESeq2 pipeline ^22^. Genes were filtered by an RPKM threshold greater than 3. Differential gene expression (DGE) analyses were performed using DESeq with a parametric fit and iterative size factor estimation. Results were filtered using a p-value cutoff of 0.01 and LFC shrinkage was applied using apeglm ^23^. A variance-stabilizing transformation was applied and genes with a minimum absolute Log2FC exceeding 0.5 were included in downstream analysis. Principal component analysis was performed on the variance-stabilized and filtered results using prcomp. A scree plot indicated principal component 1 accounted for ~ 98% of the variance. In order to identify a network of highly differentiating genes, the genes with loadings in the top and bottom 10% of principal component 1 and those with loadings in the top and bottom 15% of principal component 2 were included in network analysis.

The differentially expressed genes with a minimum absolute Log2FC exceeding 0.5 were subsequently used in network construction by entering them into the StringApp in Cytoscape ^24^. The filters for drawing edges between each gene pair were set at a total confidence score of 0.5 and a minimum co-expression score of 0.2 in order to generate a gene co-expression network.

### 2.8 Western blot analysis

Cell extracts from cultured cells were denatured by bringing them to 95°C for 5 min with 4× sodium dodecyl sulfate (SDS) loading buffer in proportion. Denatured protein (20 mg) was loaded and separated on an 8–16% SDS–polyacrylamide gel. After electrophoresis, the iBlot 2 Dry Blotting System (Thermo Fisher Scientific, IB21001) and iBind Western System (Thermo Fisher Scientific, SLF2000) were used to transfer proteins to a polyvinylidene fluoride (PVDF) membrane for analysis with antibodies. Blots were developed using SuperSignal^™^ West Pico PLUS Chemiluminescent Substrate (Life Technologies, 34580).

### 2.9 Statistics

Statistical analyses were performed using GraphPad Prism 10 (GraphPad Software, Inc). Data normality was determined using the D’Agostino-Pearson and Shapiro-Wilk tests. Data with normal distributions were analyzed using unpaired Student’s t tests (data with 2 groups), ordinary one-way ANOVAs (data with > 2 groups), and two-way ANOVAs (data with multiple variables). Normal data with significantly different variances were analyzed with Welch’s correction. Data with non-normal distributions were analyzed using Mann-Whitney U tests (data with 2 groups) and Kruskal-Wallis tests (data with > 2 groups). Post hoc multiple comparison tests were selected based on the recommendation of GraphPad Prism 10: Tukey’s test to compare every mean with every other mean, Dunnett’s test to compare a control mean with the other means, Šídák’s test to compare the means of prespecified pairs of columns, and Dunn’s test for non-normal data. Pairing and repeated measures were applied when appropriate. Suspected outliers were tested by the ROUT method (Q = 1%), and—once identified—were excluded from further analysis. Non-linear regression was used to determine if one curve adequately represents two data sets using the best-fit values (Y0, Plateau, K). Significance was determined by a p-value of ≤ 0.05, indicated with asterisks. Statistical analyses, post hoc tests, and p-values are all described in corresponding figures and figure legends.

## 3. RESULTS

### 3.1 ALMS1 protein is depleted in the proband fibroblasts

We previously conducted whole-exome sequencing (WES) analyses on ten pEFE families and identified protein-damaging variants in *ALMS1* in three patients with pEFE. We selected the current proband for in-depth mechanistic studies based on parental consent, the availability of explanted heart tissues and patient-derived fibroblasts, and the subsequent clinical follow-up at our institution. From trio-WES analyses of the proband and both parents, we identified a novel homozygous variant [c.1943delA] in *ALMS1*, as a candidate causal gene for the pEFE phenotype ^7^.

ALMS1 is a large protein (4169 AA; 461.2 kDa) whose C-terminal ALMS motif [aa: 4032–4164 (Ensembl)] is required for targeting ALMS1 to the centrosome and basal body of cilia ^25^. Importantly, the novel *ALMS1* variant [c.1943delA] resulted in a frameshift [p. Val649fs] mutation, leading to premature termination and loss of the entire ALMS motif ^7^. As predicted, we confirmed ALMS1 protein localization to the centrosomes throughout the cell cycle in the control *ALMS1*^*+/+*^ human neonatal dermal fibroblasts (hNDFs), while it is not detectable in the proband *ALMS1*-variant dermal fibroblasts. Importantly, the loss of ALMS1 in proband fibroblasts is accompanied by significant spindle elongation and chromosomal lagging in metaphase [Fig. 1A–E]. Similarly, we confirmed ALMS1 localization at the basal body of cilia in the control hNDFs, while it is not detectable at the proband basal body [Fig. 1F]. Together, these data indicate that the *ALMS1* variant abolishes ALMS1 localization at centrosomes in proband cells and is associated with spindle abnormalities and defects in chromosome alignment at the middle of the cell in metaphase.

### 3.2 ALMS1 loss induces a myofibroblast-like phenotype in proband fibroblasts.

We previously showed that ALMS1 loss alters the molecular and functional properties of proband fibroblasts, including decreased proliferation activity, enhanced migration capacity, and significant transcriptome changes compared to control cells ^7^. Aiming to identify potential molecular players driving the phenotype, we mined RNA-sequencing data derived from *ALMS1*-variant proband fibroblasts and control hNDFs for subsequent network analysis.

**Figure 2:**
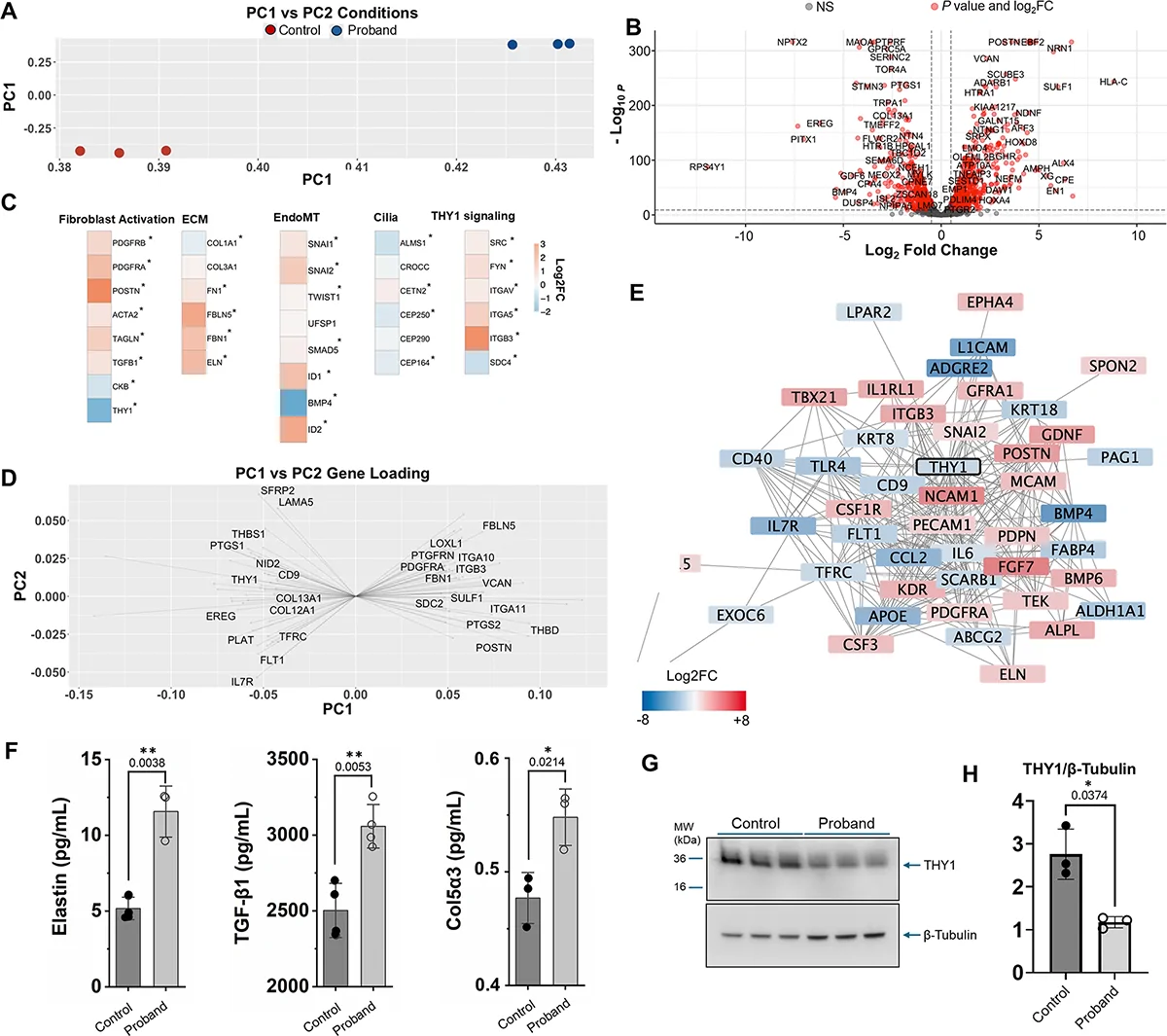
ALMS1 loss alters transcriptome programming in proband fibroblasts. (**A**) RNA-seq data of *ALMS1-*variant fibroblasts (proband) versus *ALMS1*^*+/+*^ hNDFs (control). N=3 replicates/sample. Principal component analysis of the top 1000 varied genes plotted on principal component 1 (PC1) versus principal component 2 (PC2). (**B**) Volcano plot showing significantly differentially expressed genes with the new filtering criteria of bulk RNAseq data. (**C**) Changes in expression of genes related to fibroblast activation, ECM production, EndoMT, and cilia structure are shown, with (*) indicating p-value < 0.01 between control and proband dermal fibroblasts. (**D**) Loadings of the top 10% and 15% along PC1 and PC2, respectively, plotted in PC1 versus PC2. Longer lines indicate more influence on PC1. (**E**) Network of dysregulated genes in proband fibroblasts, including *THY1*. All genes with loadings in the top 10% and 15% along PC1 and PC2, respectively, that have a connection to another gene as defined by StringApp database (Cytoscape) are shown. Color indicates Log2FC versus control, all of which are significant with **p< 0.01. Red: upregulated, Blue: downregulated in the DEGs of control vs proband fibroblasts. THY1 is highlighted by a black box. (**F**) ELISA analysis of TGFβ1, elastin, and COL5A3. (**F, G**) Western blot analysis and quantification of THY1 protein expression in control hNDFs and proband fibroblasts. Statistics for panels F-G were calculated using unpaired Student’s t-tests. Error bars: Mean±SD; RPKM: Reads per KB per Million Mapped Reads. **p< 0.01. ***p< 0.005.

Principal component analysis of the top 1000 differentially expressed genes (DEGs) revealed that control and proband fibroblasts form distinct clusters and separate along principal component 1 (PC1), capturing 98% of the variance [Fig. 2A; **SuppFig.1**]. In total, 3900 DEGs were identified in proband versus control fibroblasts [*P value* ≤ 0.01], suggesting global transcriptional reprogramming [Fig. 2B]. The upregulated genes were enriched with endoMT gene ontology terms, including myofibroblast activation markers (*ACTA2*, *TAGLN, POSTN, PDGFRA*), key signaling players of endoMT (*TGFB1*), and ECM remodeling (*ELN, FBN1*). In contrast, cell cycle activities and cilia-related terms were enriched in the downregulated genes [Fig. 2C; **SuppFig.2**]. KEGG pathway analysis revealed TGFβ among the top-enriched signaling pathways, with DEGs involved in endoMT, fibroblast activation, ECM production, and ciliary structure [Fig. 2B; **SuppFig.2]**.

Genes with loadings in the top 10% along PC1 and top 15% along PC2 driving the variance between the proband and control fibroblasts are plotted in Fig. 2D. Among these genes, we identified *THY1*, also known as CD90, as being downregulated in the proband fibroblasts. THY1 is a glycoprotein on the surface of fibroblasts that decreases during fibroblast activation ^26–28^. Indeed, in proband fibroblasts, *THY1* is negatively associated with markers of fibroblast activation, including *POSTN*, elastin (ELN), and elastin-associated ECM proteins (*FBLN5* and *FBN1*), all of which were upregulated [Fig. 2D], leading to our interest in this protein. Further, StringApp in Cytoscape ^24^, a resource that graphically shows experimentally derived connections between genes with evidence of co-expression, revealed significant connectivity between THY1 and the other genes in the fibroblast activation network [Fig. 2E].

Consistent with RNA-seq data, ELISA assays revealed increased production of TGFβ1, ELN, and COL5A3 proteins in proband fibroblasts [Fig. 2F], while THY1 protein levels were decreased [Fig. 2G,H]. Collectively, the transcriptomic signature, network analysis, and protein levels reflect the hallmarks of pEFE pathology, including endoMT induction, TGFβ activation, myofibroblast marker gene expression, and elastin-associated ECM protein production.

**Figure 3:**
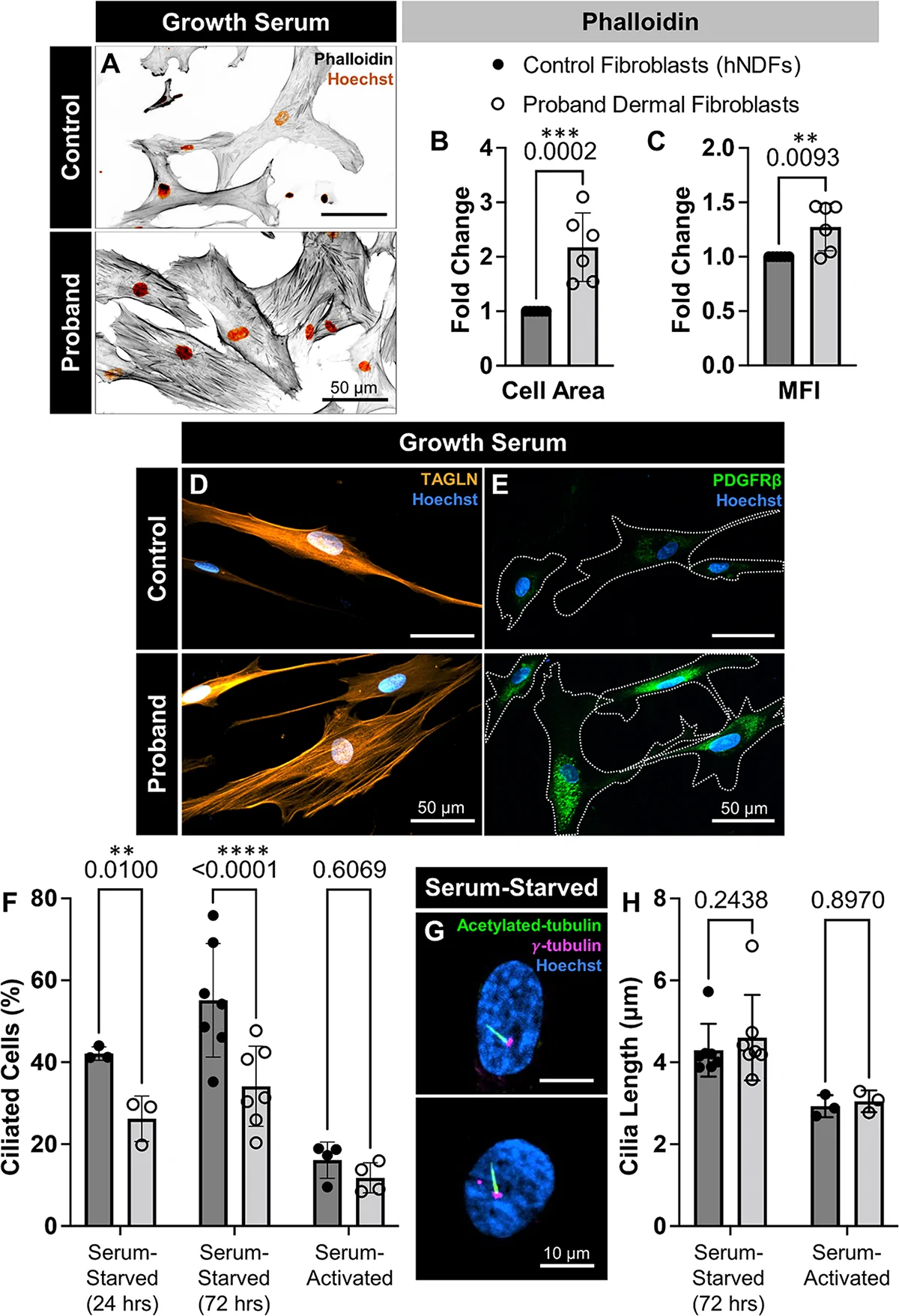
ALMS1 loss induces a myofibroblast-like phenotype and decreases ciliation in neonatal dermal fibroblasts. (**A-C**) Representative images of staining of actin filaments (**A**; phalloidin) and quantification of the cell surface area and phalloidin intensity (MFI) (**B,C**). Immunostaining of TAGLN (**D**) and PDGFRβ (**E**) in control hNDFs and proband fibroblasts. Proband cells display an increase in filamentous actin networks, cell size, and PDGFRβ expression compared to control fibroblasts. **(F)** Quantification of percent ciliated cells. Fewer proband fibroblasts are ciliated after 24 and 72 hours in serum-starved (stalled in G_0_) conditions compared to control fibroblasts. The addition of growth serum after serum-starvation (i.e. serum-activated; 48 hours serum-starvation followed by 24 hours growth serum) induces re-entry into the cell cycle and cilia disassembly. Serum-activated proband fibroblasts display a similar number of ciliated cells compared to control fibroblasts, suggesting unimpaired cilia disassembly. **(G)** Immunostaining of cilia (acetylated-tubulin) and basal bodies (*γ*-tubulin). The cilia in control versus proband cells are equivalent in length in serum-starved (G_0_) and serum-activated (proliferative) conditions, indicating that while there are fewer cilia in G_0_ proband fibroblasts, the cilia that are present can assemble and disassemble to comparable lengths. Cilia length quantifications are shown in **H**. Cell Area (**B**) and MFI (**C**) are used in Figure 6 to compare additional data, thus, p-values between control and proband samples were determined from two-way ANOVAs with Šídák’s multiple comparisons test. Statistics for panels G and I were calculated using two-way repeated measures ANOVAs with Šídák’s multiple comparisons test. Each data point represents an independent experiment. Data are presented as the mean ± S.D. Scale bars: 50 μm (**A,D,E**), 10 μm (**H**).

To investigate whether *ALMS1*-variant proband fibroblasts display morphological features of myofibroblast differentiation, we analyzed the actin cytoskeleton and cell size of proband fibroblasts versus control hNDFs. Phalloidin staining revealed that proband cells exhibit enhanced filamentous actin networks and increased cell surface area, which is consistent with a myofibroblast-like phenotype [Fig. 3A–C]. Likewise, immunostaining of transgelin (TAGLN; also known as SM22α), a TGFβ-inducible actin-binding protein ^29,30^, indicated increased filamentous actin and cell surface area [Fig. 3D]. In addition, PDGFRβ levels are increased in proband cells [Fig. 3E]. PDGFRβ signaling has been shown to contribute to fibroblast activation ^31,32^; thus, the increased cell size, filamentous actin, and PDGFRβ expression in proband cells support a shift toward a myofibroblast-like state. Altogether, these data indicate that *ALMS1* loss induces fibroblast activation, altering the proband fibroblast towards a myofibroblast-like phenotype.

### 3.3 ALMS1 loss reduces ciliation in proband fibroblasts.

As ALMS1 has been implicated in ciliogenesis ^8–10^, we next analyzed the cilia in proband versus control fibroblasts. After 24 hours of serum starvation to stall cells in G_0_ and induce cilia assembly, proband fibroblasts were less ciliated than control fibroblasts [Fig. 3F]. Since proband dermal fibroblasts proliferate at a slower rate than control fibroblasts ^7^, we increased the serum-starvation time to 72 hours to ensure all cells had reached G_0_, which similarly showed a significant decrease in the percentage of ciliated proband cells [Fig. 3F].

To determine if proband fibroblasts experience impaired cilia disassembly, we added growth serum after serum starvation (i.e., serum-activated) to induce re-entry into the cell cycle and promote cilia disassembly and shortening. Serum-activated proband fibroblasts display a similar percentage of ciliated cells compared to control fibroblasts, suggesting normal cilia disassembly [Fig. 3F]. Next, we analyzed the lengths of cilia in proband and control fibroblasts in serum-starved and serum-activated conditions. The cilia in proband cells are comparable in length to those in control fibroblasts [Fig. 3G,H], indicating that while there are fewer cilia in G_0_ proband fibroblasts, the cilia that are present can assemble and disassemble to comparable lengths.

### 3.4 ALMS1 forms a “bull’s-eye” cap on the proximal end of the centriole, and ALMS1 loss disrupts the organization of proximal centriolar linker proteins.

**Figure 4:**
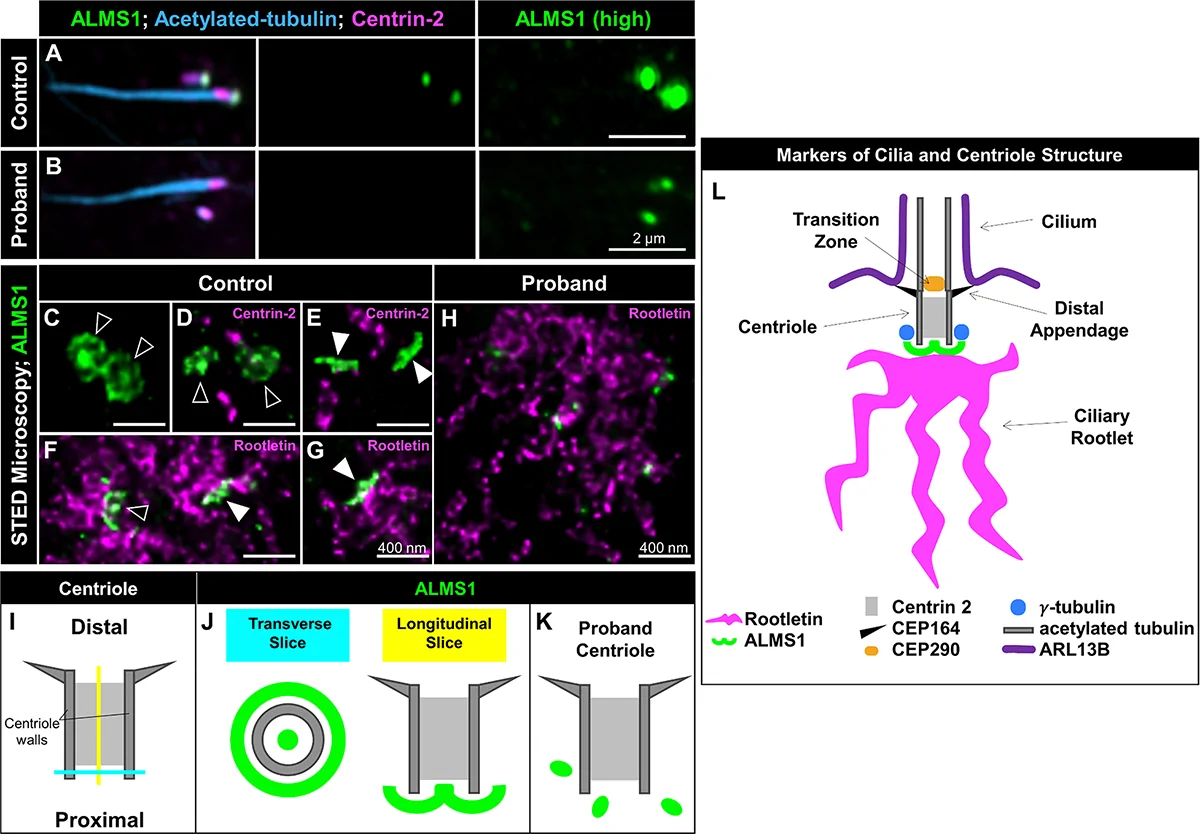
ALMS1 forms a cap with a concave center on the proximal end of the centriole and is fragmented in proband cells. (**A,B**) Immunostaining of cilia (acetylated-tubulin), centrioles (Centrin-2), and ALMS1 in control (**A**; hNDFs) and proband (**B**) fibroblasts. Imaging using Zeiss 880 Airyscan microscopy reveals a profound loss of ALMS1 protein at the proximal end of the centriole in proband fibroblasts, although increased (high) exposure times reveal retention of a small amount of appropriately localized ALMS1 protein. **(C-H)**STED super-resolution microscopy with ALMS1, Centrin-2, and Rootletin in control (**C-G**) and proband (**H**) fibroblasts. White arrows indicate ALMS1 imaged longitudinally, and black arrows indicate transverse imaging. ALMS1 appears as a centriolar cap with a concave center in control fibroblasts and as small protein fragments in proband fibroblasts. **(I-K)** Schematic rendering of ALMS1 cap structure on control and proband centrioles. A transverse section of ALMS1 on the proximal end of the centriole appears as a “bull’s-eye,” while a longitudinal section through the centriole appears as a “w”, as seen in **J**. The ALMS1 fragments in proband centrioles appear as small, uncoordinated spots near the proximal end of the centriole (**K**). (**L**) Schematic rendering of the cilia and centriolar structural proteins imaged in this study. Scale bars: 2 μm (A,B), 400 nm (C-H).

Confocal imaging with a Zeiss LSM 880 with Airyscan microscope reveals that proband fibroblasts experience a profound loss of ALMS1 protein at the proximal end of the centriole, although increased (high) imaging exposure times reveals retention of a small amount of ALMS1 protein at this site [Fig. 4A,B]. Additional imaging of control fibroblasts via stimulated emission depletion (STED) super-resolution microscopy shows that ALMS1 forms a distinct cap with a concave center (“bull’s-eye”) at the proximal end of the centriole in control fibroblasts [Fig. 4C–G,I,J]. In proband cells, ALMS1 localization is fragmented, with only a small amount of protein detectable near the proximal end of the centriole [Fig. 4H,K]. A diagram of cilia and centriole structural proteins used for imaging is shown in Fig. 4L.

**Figure 5:**
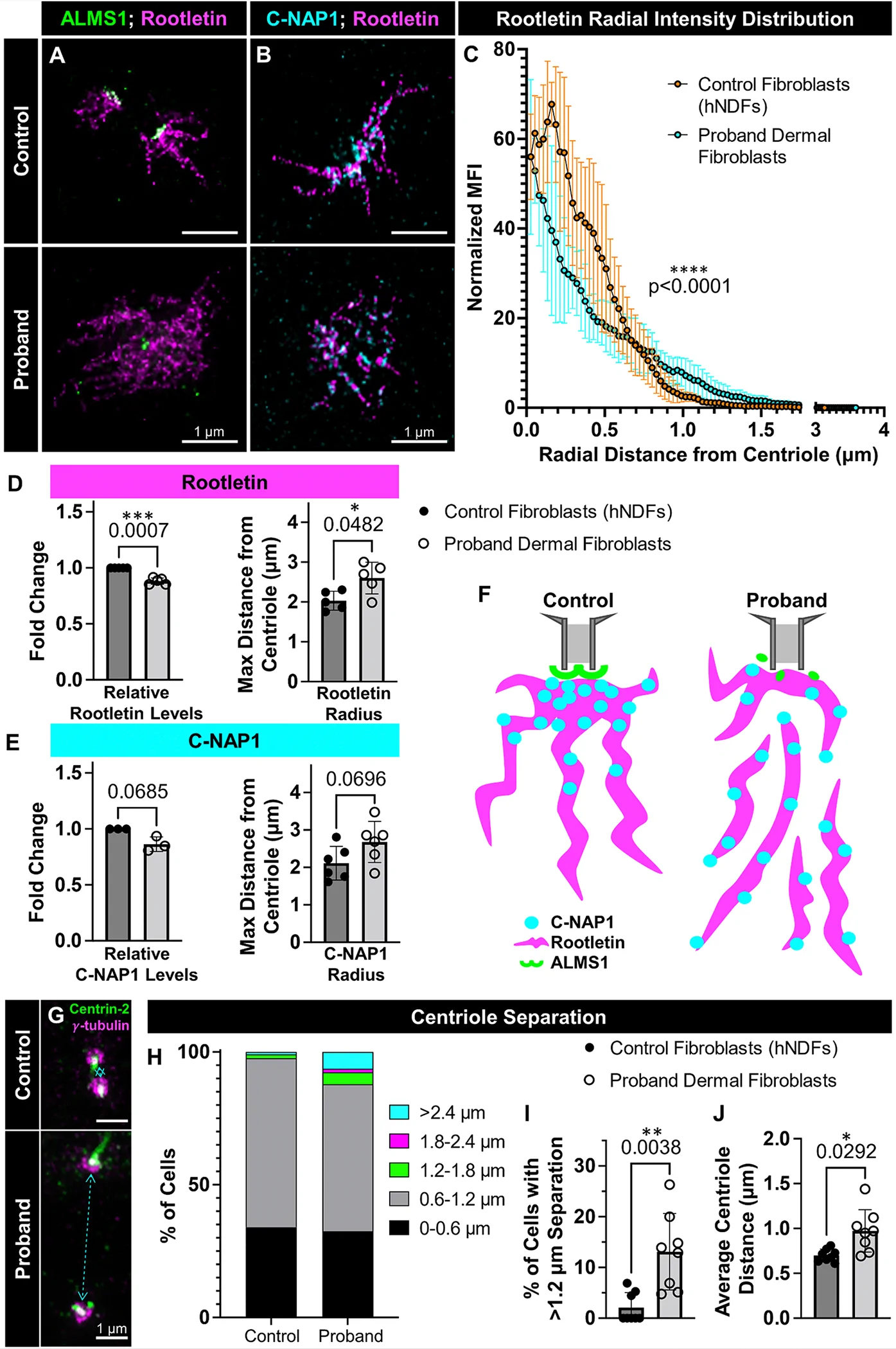
ALMS1 loss untethers the Rootletin network and C-NAP1 from the centriole and increases the likelihood of severe centriole separation. (**A-D**) STED super-resolution imaging of Rootletin and ALMS1 (**A**) or C-NAP1 (**B**) in control (hNDFs) and proband fibroblasts. Proband cells display disorganized and more dispersed Rootletin than control fibroblasts. C-NAP1, which intercalates into Rootletin fibers, also appears more dissociated in proband cells. **(C)** A radial intensity distribution plot showing the mean fluorescence intensity (MFI) of Rootletin from the centriole to the outer radius of Rootletin fibers. Control fibroblasts (orange) display a higher MFI at regions closest to the centriole, whereas proband fibroblasts (cyan) show lower Rootletin intensity near the centriole, with MFI persisting for a longer radial distance. This quantitatively suggests that Rootletin is more dispersed in proband fibroblasts. **(D,E)** The relative levels and maximum radius of Rootletin (**D**) and C-NAP1 (**E**). To calculate relative levels, total fluorescent signal for Rootletin/C-NAP1 was thresholded using Fiji Analysis software, then measured for total area covered and MFI. Integrated density, which is (total area) × (MFI), is reported as “Relative Levels.” There is a minor yet significant decrease in the relative Rootletin levels, and a minor downward trend in relative C-NAP1 levels in control versus proband fibroblasts, suggesting that the amount of protein at the centriole is slightly decreased. Maximum radius was calculated using the max distances reported from the radial intensity distribution plot generated with Fiji. The maximum radii are increased, further indicating that Rootletin and C-NAP1 are more dispersed. **(F)** Schematic rendering of Rootletin and C-NAP1 organization around the centriole in control and proband fibroblasts. **(G-J)** STED super-resolution imaging of Centrin-2 and *γ*-tubulin to identify centrioles. The distance between centrioles was measured, and the percentage of cells with centrioles separated by the indicated distances was plotted in **H**. Proband fibroblasts display an increase in the percentage of cells with moderate or severe centriole separation (>1.2 μm; **I**), with a complementary increase in the average distance between centrioles (**J**). Statistics for panel C were calculated using nonlinear regression to determine if one curve adequately represents the two data sets using the best-fit values (Y0, Plateau, K). A p-value of <0.0001 indicates that the data are represented by two significantly different curves [F(3,647) = 71.29; n=3 independent experiments]. Statistics for panels D and E (Relative Levels) and J were calculated using unpaired t-tests with Welch’s correction, and for panel E (C-NAP1 Radius) they were calculated using a paired t-test. One outlier was identified and removed from Relative Rootletin Levels (**D**). Rootletin Radius (**D**) and Average Centriole Distance (**J**) are used in Figure 7 to compare additional data, thu,s p-values between control and proband samples were determined from two-way ANOVAs with Šídák’s multiple comparisons test. Each data point in D, E, I, J represents an independent experiment. Data is presented as the mean ± S.D. Scale bars: 1 μm.

We next examined the consequences of ALMS1 loss on centriolar architecture using STED super-resolution microscopy. In control fibroblasts, Rootletin—which is involved in anchoring centriolar pairs together ^33^—forms a fibrous network anchored by ALMS1 at the proximal end of the centriole. This network is untethered and disorganized in proband cells [Fig. 5A]. In control fibroblasts, C-NAP1—the ALMS1-Rootletin bridging protein ^34,35^—localizes near the centriole proximal end in a punctate pattern and intercalates within the Rootletin fibers [Fig. 5B; **SuppFigure 3A-I**]. Like Rootletin, C-NAP1 appears more disordered in proband cells [Fig. 5B]. Quantification of Rootletin mean fluorescence intensity (MFI) in relation to its radial distance from the centriole reveals that Rootletin is concentrated closer to the centriole in control cells than in proband cells [Fig. 5C,D]; the same trend is seen for C-NAP1 [Fig. 5E]. Furthermore, the relative protein levels (as determined by integrated density, calculated from area × MFI) of Rootletin and C-NAP1 are modestly decreased [Fig. 5D,E]. Together, these data indicate that the amount of Rootletin and C-NAP1 protein at the centriole is decreased, while the maximum radial dispersion of both is increased in proband cells compared to controls, suggesting impaired interactions between the mother and daughter centrioles. These changes are diagrammed in Fig. 5F.

To evaluate coordination between centriole pairs, we assessed the degree of centriole separation in control and proband fibroblasts in G_0_, following serum starvation. Proband fibroblasts display an increase in the percentage of cells with moderate or severe centriole separation [> 1.2 μm; Fig. 5G–I], with a complementary increase in the average distance between centrioles [Fig. 5J]. This suggests that the loss of ALMS1 and the associated disruption of Rootletin and C-NAP1 organization promotes aberrant centriole separation. Notably, immunostaining analysis for distal centriole markers CEP164 and CEP290 indicates that distal centriole structures remain intact in proband cells [**SuppFig. 4A-E**], suggesting that ALMS1 loss selectively affects proximal centriole structure.

### 3.5 sTHY1 rescues ALMS1-dependent myofibroblast-like phenotypes but not cilia and centriolar structural phenotypes.

**Figure 6.**
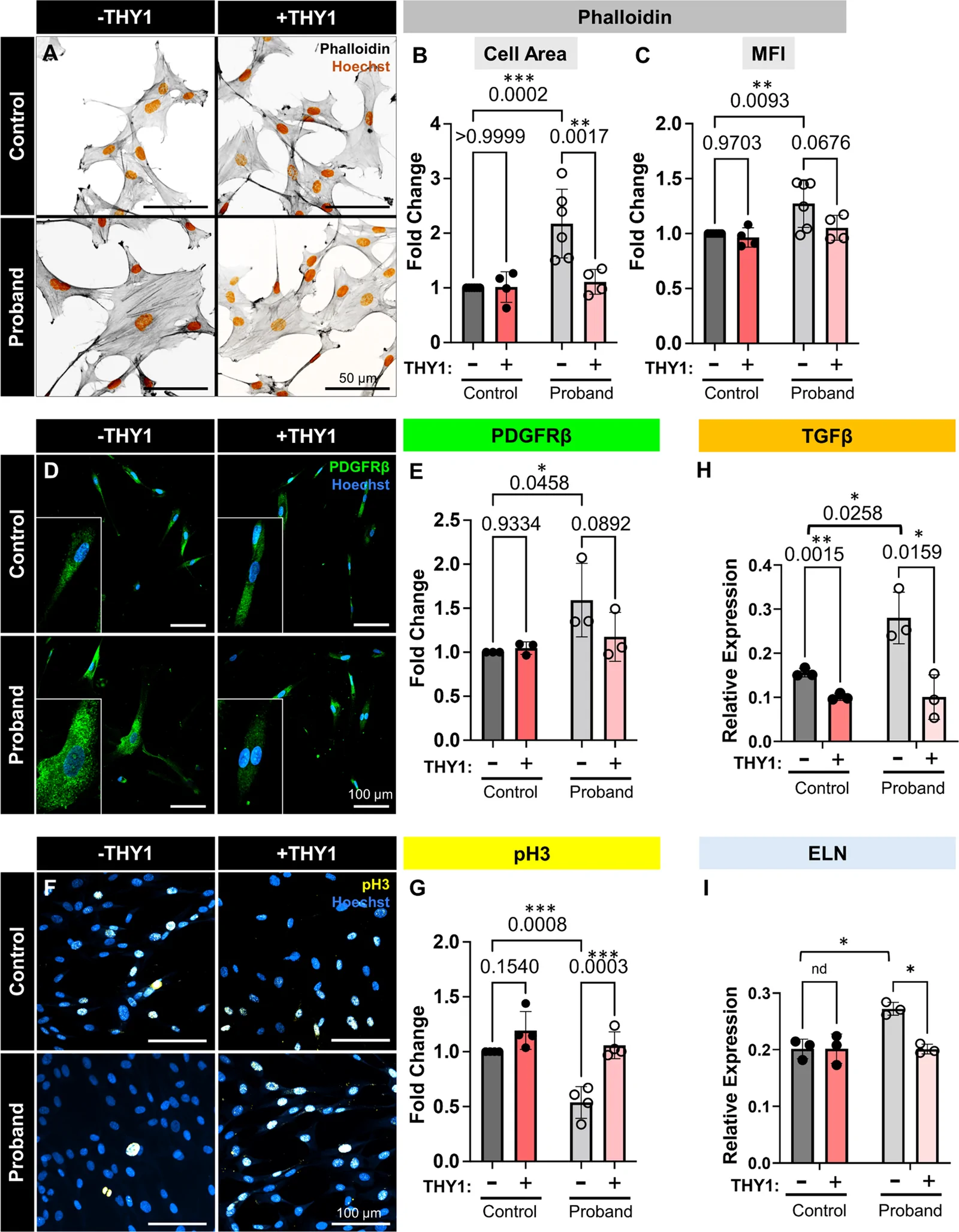
sTHY1 treatment rescues the myofibroblast-like phenotype of proband fibroblasts. (**A**) ** Staining of actin filaments (phalloidin) in control and proband fibroblasts with or without sTHY1. (**B,C**) Quantification of the cell area (**B**) and mean fluorescence intensity (**C**; MFI) of phalloidin-stained cells with or without sTHY1. Proband cells display increased cell size and phalloidin intensity, which is rescued with THY1 treatment. The control and proband phalloidin data without sTHY1 are the same data presented in Figure 3C,D. (**D**) Immunostaining of PDGFRβ ** in control and proband fibroblasts with or without sTHY1. (**E**) ** Quantification of the MFI of PDGFRβ-stained cells with or without sTHY1. ** Proband cells display increased PDGFRβ expression, which is rescued with Thy1 treatment. (**F**)pH3 Immunostaining (proliferation marker) in control and proband fibroblasts with or without sTHY1. (**G**) Quantification of the percentage of pH3+ nuclei in cells with or without sTHY1. Proband cells display significantly fewer pH3+ nuclei. sTHY1 induces a modest increase in proliferation in control cells but significantly rescued the impaired proliferation in proband cells back to levels comparable to the control. All quantifications are reported as Fold Change compared to control. (**H-I**) Quantitative real time PCR (qPCR) analysis of *TGFB1* (**H**) and *ELN*(**I**) in control and proband fibroblasts with or without sTHY1. Proband cells display increased *TGFB1* and *ELN* expression, which is reversed after sTHY1 treatment. Cells were treated with sTHY1 (1 μg/mL) or vehicle control for 48 hours in the presence of growth serum for phalloidin, PDGFRβ, pH3, and qPCR analysis. Statistics for panels H and I were calculated using unpaired Student’s t-tests. Statistics for panels B, C, and G were calculated using two-way ANOVAs with Šídák’s multiple comparisons test. Statistics for panel E were calculated using a two-way repeated measures ANOVA with Šídák’s multiple comparisons test. Each data point represents an independent experiment. Data are presented as the mean ± S.D. Scale bars: 50 μm (A), 100 μm (D,F).

To assess whether phenotypic defects of proband fibroblasts could be rescued, we treated proband and control fibroblasts with soluble THY1 (sTHY1). sTHY1 treatment restored the cell size and actin filament networks in proband cells to near-control levels [Fig. 6A–C] and rescued PDGFRβ expression [Fig. 6D,E]. Furthermore, pH3 staining revealed that the decrease in cell proliferation previously observed in proband fibroblasts ^7^ was restored to control levels with sTHY1 treatment [Fig. 6F,G]. sTHY1 treatment was also able to rescue the increased transcript levels of *TGFB1* and *ELN* [Fig. 6H,I]. Together, these data indicate that the proband myofibroblast-like morphology and markers, in addition to proliferation levels, are largely rescued by sTHY1 treatment.

**Figure 7:**
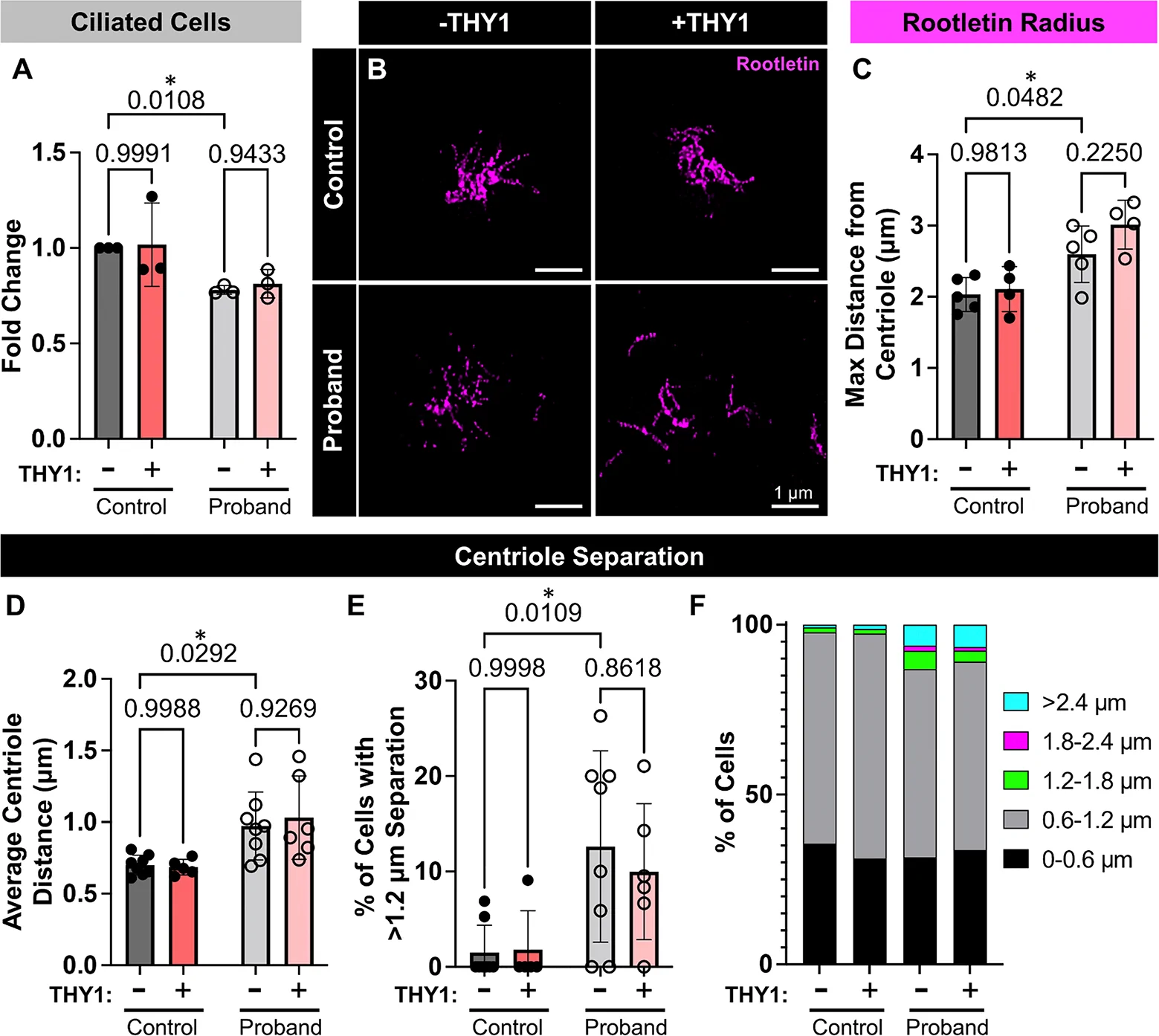
sTHY1 treatment does not rescue the disrupted organization of the centriolar material. (**A**) Quantification of percent ciliated cells (reported as Fold Change) with or without sTHY1. sTHY1 treatment did not rescue the number of ciliated proband cells. (**B**) Immunostaining of Rootletin in control and proband fibroblasts with or without sTHY1. (**C**) Quantification of the maximum radius of Rootletin signal. The control and proband data without sTHY1 are the same data presented in Figure 5D. The increased radius of Rootletin in proband cells was not rescued by sTHY1 treatment, suggesting that sTHY1 does not impact the disorganized centriolar material caused by ALMS1 loss. (**D-F**) Quantification of the average distance of centriole separation (**D**), the percentage of cells with >1.2 μm centriole separation (**E**), and the percentage of cells with centrioles separated by the indicated distances (**F**) with or without sTHY1. The control and proband data without sTHY1 in **D** are the same data presented in Figure 5J. Proband fibroblasts display an increase in the average distance between centrioles and percent of cells with moderate to severe separation, which is not rescued by sTHY1 treatment. Cells were treated with sTHY1 (1 μg/mL) or vehicle control for 48 hours in serum-starvation conditions for analysis of cilia, the centriolar material, and centriole separation. Statistics for panel A were calculated using a two-way repeated measures ANOVA with Šídák’s multiple comparisons test. Statistics for panels C, D, and E were calculated using two-way ANOVAs with Šídák’s multiple comparisons test. One outlier was identified and removed from panel D. Each data point represents an independent experiment. Data are presented as the mean ± S.D. Scale bars: 1 μm.

Treatment with sTHY1, however, did not restore proband cell ciliation nor the disrupted organization of Rootletin at the centriole [Fig. 7A–C]. Consistent with this, average centriole distance and the percentage of cells with severe centriole separation were not rescued by sTHY1 treatment [Fig. 7D–F]. These data suggest that while sTHY1 can regulate the myofibroblast-like phenotypes and restore proliferation defects that arise downstream of ALMS1 loss, it does not rescue the disorganized centriolar material or reduced ciliation.

## 4. DISCUSSION

Here, we show that ALMS1 loss in *ALMS1*-variant proband fibroblasts leads to reduced ciliation and disruption in proximal centriolar architecture. These defects are accompanied by global transcriptome reprogramming, protein-level changes, and morphological features characterized by actin remodeling and increased PDGFRβ expression, collectively indicating a fibroblast transition toward an activated, myofibroblast-like phenotype. Importantly, we find that *THY1*, a fibroblast cell surface marker that is downregulated in activated fibroblasts (myofibroblasts), is also significantly downregulated in our *ALMS1-*variant proband fibroblasts. Notably, sTHY1 treatment rescues these myofibroblast activation-associated phenotypes without restoring ciliary or centriolar defects.

As part of our analysis, we report the first STED-resolution view of wild-type ALMS1 protein at the proximal end of the centriole. Previously reported as a cap-like structure ^25^, we find that it contains a concave center that extends slightly into the centriole lumen. We also show via super-resolution STED imaging that C-NAP1 proteins do not coalesce to form a single continuous structure ^34,35^ on the proximal end of centrioles in dermal fibroblasts but appear as punctate regions intercalated into Rootletin fibers with a concentrated signal at the centriole interface.

Our previous analysis of the proband predicted a novel homozygous loss-of-function variant within *ALMS1* ^7^. Our traditional immunostaining assays indicated that this genetic variant resulted in a null mutation ^7^; however, our STED super-resolution imaging has revealed that there may be a small amount of truncated ALMS1 protein present, suggesting that the proband variant may be a hypomorphic allele. As the *ALMS1* proband variant [c.1943delA] results in a frameshift and premature stop codon within the first 16% of the full-length *ALMS1* amino acid sequence, we speculate that truncated ALMS1 fragments may be produced but execute an inefficient function. The [c.1943delA] variant leads to a loss of the ALMS motif, which is thought to play an essential role in targeting the ALMS1 protein to the centrioles and basal body of cilia ^25^. However, a previous study did show that the N-terminus of ALMS1 is sufficient for cilia formation in the mouse kidney ^10^, raising the possibility that more complex interactions could exist between fragments of ALMS1 and the proximal end of the centriole.

Past studies on Alström syndrome patients and models of *ALMS1* loss in animals and cell culture systems have revealed a variety of cilia or centriolar defects that are not consistent among models and mutations. For instance, the ability to form primary cilia appeared normal in all three *Alms1*-deficient mouse models reported to date; however, an age-dependent loss of cilia was observed in two out of the three studied models ^19^. Thus, the exact ciliary and centriolar phenotypes present in AS patients may be cell-specific, mutation-based, or age-dependent. While previous reports have noted phenotypes such as cilia shortening ^9,10^, lengthening or bending ^8^, and centriole duplication defects due to ALMS1 loss ^11^, these phenomena were not observed in our proband dermal fibroblasts. This is consistent with the possibility that our identified variant in the proband cells leads to a small amount of truncated ALMS1 protein that potentially retains partial function, as the cilia that do form appear morphologically normal, despite the disorganization of fibers at the proximal ends of the centrioles.

In line with this, we do see defects in centriole connection, as indicated by a decrease in Rootletin and C-NAP1 levels, an increase in radial dispersion of these networks, and a moderate-to-severe centriole separation defect. A similar centriole separation phenotype has been reported in ALMS1 knockdown cells ^25^. Defects in centriole connectivity can—though not always—lead to ciliogenesis defects ^36,37^. Since the cilia that form in proband dermal fibroblasts are equivalent in length and morphology to control fibroblasts, the underlying driver of decreased ciliation in these cells remains unclear. Given that ciliation is tightly linked to the cell cycle, altered proliferation rates in proband dermal fibroblasts may contribute to the reduced percentage of ciliated cells. However, the inability of sTHY1 to rescue ciliation, despite rescuing proliferation, suggests that additional mechanisms are involved.

Our data also raises the possibility that ALMS1-mediated disruption of centriole organization and ciliogenesis may contribute to altered fibroblast signaling, activation, and proliferation. Indeed, primary cilia are essential signaling hubs for several pathways including TGFβ and PDGFR ^14^. We previously reported that proband fibroblasts experience increased TGFβ signaling ^7^, while others have shown that ALMS1 loss in cell culture led to downregulation of TGFβ signaling ^8,38^. As part of our current work, we show that THY1, an inhibitor of TGFβ, is suppressed in proband fibroblasts. Indeed, loss of THY1 is strongly correlated with an increase in TGFβ1, elastin, and elastin-associated ECM proteins in the proband cells.

THY1 treatment has been shown to attenuate cardiac fibroblast activation in response to stress by inhibiting TGFβ, SMAD3, and COL1A1^27^. Similarly, *THY1* loss is correlated with lung fibrosis in response to stress, and the addition of sTHY1 has been shown to rescue mouse lung fibrosis ^28,39^. These data suggest that the decreased THY1 in our proband fibroblasts may drive the myofibroblast-like phenotype underlying the pEFE pathology. Indeed, sTHY1 may rescue the myofibroblast-like phenotypes in our proband cells by promoting cell proliferation and re-entry into the cell cycle—although the exact mechanisms driving this response require further investigation. The ability of sTHY1 to rescue fibroblast activation without overtly restoring ciliary or centriolar defects suggests that these phenotypes arise through separate downstream pathways following ALMS1 loss. Although it remains mechanistically unclear exactly how THY1 regulates ALMS1-mediated cellular defects, our data raises the distinct possibility that sTHY1 might be able to reverse some pEFE-related phenotypes, like the effects seen on lung fibrosis.

Along this line, our current study gives us insight into the altered expression of extracellular matrix genes in pEFE. COL1A1, generally one of the largest components of the ECM, is downregulated in pEFE, while elastin-associated glycoproteins like *FBLN5* and *FBN1* are upregulated. This increase in glycoproteins and integrins could imply altered cell-ECM interactions in proband cells ^40^; however, further studies into the composition of ECM in pEFE are needed to fully understand the implications of these changes. Interestingly, increased *FBLN5* expression is associated with myocardial dysfunction, fibrosis, and increased tissue stiffness ^41,42^, adding to our long-term interest in understanding ECM-mediated pEFE phenotypes. Moving forward, we aim to expand our pEFE patient-derived fibroblast cell lines to incorporate additional probands and variant types. This will allow us to determine patient-specific versus generalizable modalities linked to *ALMS1*-driven pEFE. Additionally, while we have taken advantage of our proband-derived fibroblasts for expression network construction, mechanistic analysis, and super-resolution imaging, our future studies will focus on validation of our findings *in vivo* using mouse models of *ALMS1* loss-of-function.

In conclusion, our data supports a model in which ALMS1 contributes to the structural integrity of the proximal centriole, is required for efficient ciliation, and helps coordinate coupling of centrioles. Disruptions to these processes in dermal fibroblasts may alter ciliary-mediated signaling, such as TGFβ, thereby promoting fibroblast activation toward a myofibroblast-like state, increasing production of elastin and elastin-associated ECM proteins, and ultimately driving a pEFE disease phenotype.

## Supplementary Material

SUPLEMENTARY DATA STATEMENT

Supplementary Data is available online.

Supplementary Files

This is a list of supplementary files associated with this preprint. Click to download.

• SupplementaryMaterial08302026.pdf

• SupplementaryFigureLegends.docx

## Figures and Tables

**Table 1:** 

Antibodies	Dilution	Source	Identifier
Rabbit polyclonal anti-ALMS1	1:50 – 1:200	Invitrogen	#PA5-103596
Rat monoclonal anti-α-Tubulin (cloneYOL1/34)	1:500	Bio-Rad	#MCA78G
Mouse monoclonal anti-Pericentrin	1:500	Abcam	#ab28144
Donkey polyclonal anti-Rat IgG (H + L), Cy3 AffiniPure	1:500	Jackson ImmunoResearch	#712–165-153
Donkey polyclonal anti-Mouse IgG (H + L), Cy5 AffiniPure	1:500	Jackson ImmunoResearch	#715–175-151
Donkey polyclonal anti-Rabbit IgG (H + L), Fluorescein (FITC) AffiniPure	1:500	Jackson ImmunoResearch	#711–095-152
mouse anti-0-SMA	1:200	Abcam	#ab7817
mouse anti-acetylated-tubulin	1:300	GeneTex	#GTX16292
rabbit anti-ALMS1	1:250	Abcam	#ab84892
rabbit anti-Arl13b	1:200	Proteintech	#17711-1-AP
rat anti-Centrin-2	1:250	BioLegend	#698602
mouse anti-CEP164	1:100	Santa Cruz Biotechnology	#sc-515403
mouse anti-CEP290	1:250	Santa Cruz Biotechnology	#sc-390462
rabbit anti-C-NAP1	1:100	Proteintech	#14498-1-AP
mouse anti-C-NAP1	1:100	Proteintech	#66814-1-IG
mouse anti-γ-NAPI	1:50	Santa Cruz Biotechnology	#sc-390540
rabbit anti-y-tubulin	1:150	GeneTex	#GTX113286
mouse anti-PDGFRβ	1:100	Abcam	#ab69506
rabbit anti-pH3	1:500	Sigma-Aldrich	#H0412
mouse anti-Rootletin	1:200	Santa Cruz Biotechnology	#sc-374056
rabbit anti-TAGLN	1:100	Abcam	#ab14106
Phalloidin 633	1:500	Abnova	#U0296
goat anti-rabbit IgG (H + L) highly cross-adsorbed secondary antibody Alexa Fluor 488	1:750	Invitrogen	#A11034
goat anti-rabbit IgG (H + L) highly cross-adsorbed secondary antibody Alexa Fluor 594	1:750	Invitrogen	#A11037
goat anti-mouse IgG (H + L) highly cross-adsorbed secondary antibody Alexa Fluor 488	1:750	Invitrogen	#A11029
goat anti-mouse IgG (H + L) highly cross-adsorbed secondary antibody Alexa Fluor 594	1:750	Invitrogen	#A11032
goat anti-rat IgG (H + L) cross-adsorbed secondary Antibody Alexa Fluor 647	1:750	Invitrogen	#A21247
goat anti-mouse STAR ORANGE	1:100	Abberior Instruments	#STORANGE-1001
goat anti-rabbit STAR ORANGE	1:100	Abberior Instruments	#STORANGE-1001
goat anti-rat STAR RED	1:100	Abberior Instruments	#STRED-1007
goat anti-rabbit STAR RED	1:100	Abberior Instruments	#STRED-1002
goat anti-mouse STAR RED	1:100	Abberior Instruments	#STRED-1001
Hoechst 34580	1 μg/mL	Sigma-Aldrich	#63493

## Data Availability

Gene expression data has been uploaded to Figshare; license CC BY 4.0. All data presented in this manuscript will be available for sharing with interested investigators.
